# Molecular Detection of *Bartonella quintana*, *B. koehlerae, B. henselae, B. clarridgeiae, Rickettsia felis*, and *Wolbachia pipientis* in Cat Fleas, France

**DOI:** 10.3201/eid0903.020278

**Published:** 2003-03

**Authors:** Jean-Marc Rolain, Michel Franc, Bernard Davoust, Didier Raoult

**Affiliations:** * Université de la Méditerranée, Marseille, France; † Ecole Nationale Vétérinaire de Toulouse, France; ‡ Conseiller Vétérinaire Régional Interarmées, Lyon Armées, France)

**Keywords:** *Bartonella*, *Rickettsia felis*, cat fleas, research

The prevalences of *Bartonella*, *Rickettsia,* and *Wolbachia* were investigated in 309 cat fleas from France by polymerase chain reaction (PCR) assay and sequencing with primers derived from the *gltA* gene *f*or *Rickettsia,* the *its* and *pap31* genes for *Bartonella,* and the 16S rRNA gene for *Anaplasmataceae.* Positive PCR results were confirmed by using the Lightcycler and specific primers for the *rOmpB* of *Rickettsia* and *gltA* of *Bartonella*. *R. felis* was detected in 25 fleas (8.1%), *W. pipientis*, an insect symbiont, in 55 (17.8%), and *Bartonella* in 81 (26.2%), including *B. henselae* (9/81; 11.1%), *B. clarridgeiae* (55/81; 67.9%), *B. quintana* (14/81; 17.3%), and *B. koehlerae* (3/81; 3.7%). This is the first report of the amplification of *B. quintana* from fleas and the first description of *B. koehlerae* in fleas from an area outside the United States. Cat fleas may be more important vectors of human diseases than previously reported.

Fleas can be found worldwide and are vectors of several important zoonoses, including plague caused by *Yersinia* *pestis* (*1*). The classic cycle of *Rickettsia typhi*, the agent of murine typhus, involves rats and the rat flea, *Xenopsylla cheopis,* the main vector (*2*). The disease is transmitted by flea bites or contact with flea feces. Recently, murine typhus has been shown to exist in some endemic foci where neither rats or their fleas are found. Subsequently, in the United States, *R. typhi* was found to be maintained in the cat flea, *Ctenocephalides felis,* collected from opossums (*2*). *R. felis* is the recently recognized agent of flea-borne spotted fever, which has been reported in various countries, including the United States, Mexico, Brazil, Germany, and France (*3*–*6*). *C. felis* is apparently the main vector of this new rickettsial disease, and *R. felis* has been found in this flea in several countries, including the United States (*2*), Brazil (*7*), Spain (*8*), and Ethiopia (*6*). A reservoir of flea-borne spotted fever in the United States may be the opossum. Of major importance to the epidemiology of the above rickettsioses is the maintenance of *R. typhi* or *R. felis* in their hosts by transovarial transmission (*9*) and the fact that neither organism is lethal for fleas.

Bartonellae are gram-negative bacteria that cause various human diseases and have various arthropods, such as lice, ticks, and fleas, as vectors (*10*). Transmission to humans may also occur by scratches or bites from reservoir hosts, especially cats. Among the genus *Bartonella*, four species have been isolated from the blood of cats. Two of these species occur worldwide, *B. henselae*, the agent of cat-scratch disease, and *B. clarridgeiae*, which might be another agent of cat-scratch disease (*11*), and two have been reported only from the United States, *B.* *koehlerae* and *B. bovis* Bermond *(B. weissii)* (*12*–*14*). The main vector of *B. henselae* infections in cats is most likely the cat flea (*2*), whereas the vectors of *B. koehlerae* and *B. weissii* are unknown. Detection of *B. clarridgeiae* in cat fleas by polymerase chain reaction (PCR) amplification has indicated the possible role of fleas as vectors of the organism (*15*). Cats are the reservoirs of the bacteria, and the prevalence of cats with *B. henselae* and *B. clarridgeiae* bacteremia ranges from 4% to 70%, according to the geographic location (*15*).

We describe experiments in which we used PCR amplification and DNA sequencing to detect *Rickettsia*, *Bartonella*, and *Ehrlichia* species in *C. felis* collected from various sites in France.

## Materials and Methods

### Source and Identification of Cat Fleas

Cat fleas (*C. felis*), identified according to current taxonomic keys (*16*), were obtained from various departments of the Veterinary School of Toulouse, located throughout France. Cat fleas were sent to our laboratory in sealed, preservative-free, plastic tubes at room temperature. To prevent contamination problems, as positive controls we used DNA from *R. montanensis* (ATCC VR-611) and *Bartonella elizabethae* F9251 (ATCC 49927), which react with the primer pairs we used in PCR but give sequences distinct from the species we were investigating. For negative controls, we used sterile water and human body lice reared in our laboratory; both negative controls were tested after every seventh cat-flea sample in our PCR.

### DNA Extraction

Fleas were immersed for 5 min in a solution of 70% ethanol/0.2% iodine, washed for 5 min in sterile distilled water and crushed individually in sterile Eppendorf tubes with the tip of a sterile pipette. Their DNA was extracted by using the QIAamp Tissue Kit (QIAGEN, Hilden, Germany) according to the manufacturer’s instructions. This kit was also used to extract DNA from the human body lice reared in our laboratory under standard conditions and used as negative controls.

### Detection of *Bartonella* spp., *Rickettsia* spp., and *Anaplasmataceae*

DNA extracts were amplified in two different runs with different target genes to confirm the results. In the first run we tested all the cat fleas by using genus-specific primers (Table 1) derived from the intergenic spacer region (*its* gene), the *pap 31* gene for *Bartonella* (*19*,*20*), the citrate synthase–encoding gene for *Rickettsia* (*17*), and the 16S RNA gene for *Anaplasmataceae* (*21*,*22*). A total volume of 2.5 μL of the extracted DNA was amplified in a 25-μL reaction mixture containing 12.5 pmol of each primer, 200 μM of dATP, dCTP, dGTP, and dTTP, and 1 U of Elongase in 1X PCR buffer with 0.8 μL of 25 mM MgCl_2_ (Life Technologies, Cergy Pontoise, France). PCR was carried out in a Peltier Thermal Cycler PTC-200 (MJ Research, Inc., Watertown, MA) with an initial 3-min denaturation step at 95°C, followed by 40 cycles of denaturation at 95°C (30 s), annealing at 50°C (30 s), and extension at 72°C (1 min). Amplification was completed by holding the reaction mixture at 68°C for 3 min to allow complete extension of the PCR products. PCR products were resolved by electrophoresis in 1% agarose gels, and when appropriately sized products were found, they were purified by using Qiagen columns (QIAquick Spin PCR purification kit; QIAGEN) before sequencing.

**Table 1:** Oligonucleotide primers used for polymerase chain reaction amplification and sequencing

Primer (reference)	Nucleotide sequence	Detected organism	References
CS-877 (*gltA* gene)	GGG GGC CTG CTC ACG GCG G	*Rickettsia* species	(17)
CS-1273 (*gltA* gene) BM59 (*rOmpB* gene) B807 (*rOmpB* gene)	ATT GCA AAA AGT ACA GTG AAC A CCG CAG GGT TGG TAA CTG C CCT TTT AGA TTA CCG CCT AA	*Rickettsia* species *Rickettsia* species *Rickettsia* species	(17) (18) (18)
URBarto1 (*its* gene)	CTT CGT TTC TCT TTC TTC A	*Bartonella* species	(19)
URBarto2 (*its* gene)	CTT CTC TTC ACA ATT TCA AT	*Bartonella* species	(19)
PAPn1 (*pap31* gene)	TTC TAG GAG TTG AAA CCG AT	*Bartonella* species	(20)
PAPn2 (*pap31* gene)	GAA ACA CCA CCA GCA ACA TA	*Bartonella* species	(20)
BartogltAForward (*gltA* gene)	TTC CGY CTT ATG GGT TTT GG	*Bartonella* species	This report
Bartokoehlerae (*gltA* gene)	AAC AAA ATA TTC ATC ATT CAG G	*B. koehlerae*	This report
Bartoclarridgeiae (*gltA* gene)	AAA GCA ATT TTT TCA AGT TCC	*B. clarridgeiae*	This report
Bartohenselae (*gltA* gene)	CAT TTC TGT TGG AAA TCC TAG	*B. henselae*	This report
Bartoquintana (*gltA* gene) EHR16SD (16S rRNA gene) EHR16SD (16S rRNA gene)	TTT TAA TGT AAT GCC AGA ATA A GGT ACC YAC AGA AGA AGT CC TAG CAC TCA TCG TTT ACA GC	*B. quintana* *Wolbachia* *Wolbachia*	This report (21) (21)

Any positive sample was tested again by using different primers and real-time PCR technology. For *Bartonella,* the forward primer of the *gltA* gene (Table 1) was used for all samples and species-specific primers targeting *B. quintana, B. henselae, B. clarridgeiae,* and *B. koehlerae* were designed as reverse primers. For *Rickettsia,* we used *rOmpB-*specific primers targeting *R. felis* (Table 1) (*18*). A real-time PCR assay was performed on DNA extracts in a Lightcycler instrument (Roche Biochemicals, Mannheim, Germany). The amplification program began with a denaturation step of 95°C for 120 sec, followed by 40 cycles of denaturation at 95°C for 15 s, annealing at 52°C for 5 sec, and extension at 72°C for 10 s with fluorescence acquisition at 54°C in single mode. Melting curve analysis was done at 45°C to 90°C (temperature transition, 20°C/s) with stepwise fluorescence acquisition by real-time measurement of fluorescence directly in the clear glass capillary tubes. Sequence specific standard curves were generated by using 10-fold serial dilutions (10^5^ to 10^6^ copies) of standard bacterial concentration of *Bartonella*.

The positive PCR products of the two runs for *Bartonella, Rickettsia,* and *Wolbachia* were sequenced by using the d-rhodamine terminator cycle-sequencing ready reaction kit (PE Applied Biosystems, Les Ulis, France) according to the manufacturer’s protocol. Sequences obtained were compared with those in the GenBank DNA database by using the program BLAST (version 2.0, National Center for Biotechnology Information; available from URL: http://www.ncbi.nlm.nih.gov).

## Results

Overall, 309 cat fleas from 92 cats from all areas of France (north, west, east, and south) were tested. Almost two thirds (60/92; 65%) of the cats lived both outdoors and indoors, 20% lived predominantly outdoors, and 15% lived exclusively indoors. Our negative controls consistently failed to yield detectable PCR products, whereas our positive controls always gave expected PCR products. We found a total of 89 fleas (28.8%) that were infected: 25 were positive for *R. felis* (25/309; 8.1%) as determined by citrate synthase–gene sequencing, and 81 were positive for *Bartonella* species (81/309; 26.2%) as determined either by *its* gene or *pap31* gene sequencing (Table 2). The sequences of the DNA amplicons we obtained were identical to those of *R. felis* (Genbank accession no. U33922), *B. henselae* (Genbank accession no. AF369527)*, B. clarridgeiae* (GenBank accession no. AF312497)*, B. koehlerae* (GenBank accession no. AF312490), or *B. quintana* (GenBank accession no. AF368391). The *Bartonella* species we identified were *B. henselae* (9/89; 11.1%)*, B. clarridgeiae* (55/89; 67.9%), *B. quintana* (14/89; 17.3%), and *B. koehlerae* (3/89; 3.7%). Our results were confirmed by a second PCR with the Lightcycler and specific primers for the *glt*A gene for *Bartonella* and *rOmp*B for *Rickettsia*. Because the primers were species-specific, we were able to demonstrate that none of the fleas contained more than one *Bartonella* species. Seventeen fleas, however, contained *R. felis* and *B. quintana* (12 fleas) or *B. clarridgeiae* (5 fleas) (Table 2). Lastly, in 55 fleas (17.8%) our *Anaplasmataceae*-specific primers amplified DNA with a sequence identical to that of *Wolbachia pipientis* (Genbank accession no. U23709).

**Table 2:** Repartition of 89 *Bartonella*-positive fleas

	*B. henselae*	*B. clarridgeiae*	*B. quintana*	*B. koehlerae*	No coinfection	Total
*Rickettsia felis*	0	5	12	0	8	25
No coinfection	9	50	2	3	17	
No. of fleas positive for *Bartonella*	9	55	14	3		81

## Discussion

*Rickettsia* and *Bartonella* infections occur worldwide and may cause serious diseases in people. Most of these pathogenic bacteria are transmitted to people by arthropod vectors such as ticks, fleas, and lice, which are also involved in the maintenance of the bacteria. The detection of these pathogenic bacteria in their vector arthropods can be used in epidemiologic studies and control strategies (*2*). We tested cat fleas from around France for the presence of *Rickettsia, Bartonella,* and *Ehrlichia* species. We found DNA of *R. felis* and various *Bartonella* species in these fleas by using PCR with primers for different specific genes and sequencing to confirm our results. All our negative controls gave no PCR products, and all fleas that tested positive were also positive with other PCRs with different target genes and different techniques.

We report for the first time the presence of *R. felis* in cat fleas from France. This bacteria has been detected previously in wild cat fleas from various countries, including the United States (*2*), Ethiopia (*6*), and, very recently, Spain (*8*), and Brazil (*7*). Since *C. felis* has a worldwide distribution and infestation with these fleas is very common, some have assumed that *R. felis* and flea-borne spotted fever should occur worldwide. We found that 8.1% of fleas from domestic cats were infected with *R. felis,* suggesting that clinical cases in humans may be prevalent in France and probably in Europe. In the United States, the infection rates of fleas, as determined by PCR amplification, have been reported to vary from 43% to 93% (*2*). Since bacteria are maintained transovarially, *R. felis* may be used as a marker to follow changes in the infection rates over time (*2*). In people, clinical cases of flea-borne spotted fever have been reported in the United States (Texas) (*3*), Mexico (*4*), France, and Brazil (*6*), and, very recently, in Germany (*5*). Preliminary serologic results indicate that flea-borne spotted fever might occur in France (*6*), and we have now shown that fleas in France are infected with *R. felis*. The disease is probably more prevalent than expected, even if the risk of transmission by fleas is unknown. Cross-reactions in serologic testing for *R. felis* are unpredictable in our experience, and thus serologic tests for *R. felis* should be performed in patients suffering from fever of unknown origin. Our findings of *R. felis* in French fleas indicate, then, that *R. felis* should be used, along with other spotted fever group rickettsiae, in serologic tests on patients suspected of having a spotted fever group rickettsiosis.

To date, *B. henselae* is the only recognized agent of cat-scratch disease; epidemiologic studies have implicated cats, which remain bacteremic for months to years, as the major reservoirs of *B. henselae* (*23*,*24*). Its DNA has been amplified from fleas found on bacteremic cats, and transmission of *B. henselae* to cats by *C. felis* has been demonstrated (*23*,*25*,*26*). Flea infestation was found to be more common in bacteremic cats than in nonbacteremic cats. The prevalence of *B. henselae* in fleas in our study was 3%, whereas *B. henselae* has been isolated from the blood of 4% to 70% of cats, depending on location, cat population, and flea infestation rate but not depending on the infection rate of fleas on the cats or the seropositivity of the cats (*15*,*25*).

Our study has confirmed that *B. clarridgeiae* may be detected in fleas, and we found a 17.8% prevalence in infected fleas, that is, 67.9% of all fleas positive for *Bartonella* by PCR. Until now, *B. henselae* was the most common species isolated from cats, and the prevalence of *B. clarridgeiae* has ranged from 16% to 30% (*25*). *B. clarridgeiae* is more prevalent in European cats than in American cats (*25*). The difference of recovery rate of *B. clarridgeiae* in these studies may be explained by the fact that the number of bacteria in the blood of cats infected with *B. clarridgeiae* is low (unpub. data) and because *B. clarridgeiae* grows more slowly in culture (*27*). Also, freezing of whole blood improves the recovery rate of *B. clarridgeiae* since bacteria are probably localized in erythrocytes (*15*).

We report, for the first time, the presence of *B. koehlerae* in cat fleas. This recently described species has only been reported in the blood of cats in the United States (*12*), and our findings support the idea that this bacteria might be transmitted by cat fleas, like other *Bartonella,* and that it has a worldwide distribution. The prevalence of this new *Bartonella* species in cats remains unknown and is probably underestimated because the bacteria are extremely fastidious and only grow on chocolate agar and not on heart infusion agar with rabbit blood, Columbia, or on sheep blood agar, which are currently widely used for the isolation of *Bartonella* (*12*).

We also report for the first time that cat fleas can contain *B. quintana*; this is surprising because, to date, the body louse was the only known vector of this species, and people are the only known natural reservoirs. Previously, however, in two clinical reports of chronic adenopathy attributed to *B. quintana* infection, the only epidemiologic risk identified was the presence of infected cat fleas (*28*,*29*). The role of the cat flea as a potential vector of *B. quintana* and its related diseases needs to be clarified, and new investigations of patients in contact with cats and their fleas are indicated. Also, we have reported cases of endocarditis due to *B. quintana* for which no epidemiologic risks (alcoholism or homelessness) were found (*30*,*31*). These findings and the fact that *B. quintana* DNA sequences can be found in ticks (*32*) indicate that other arthropod vectors apart from lice may be involved in the epidemiology of *B. quintana* infections.

Coinfection of cats with *B. clarridgeiae* and *B. henselae* has been reported, as has infection with the two different genotypes of *B. henselae*, Marseille and Houston (*25*,*33*). Recent reports suggest that interspecific competition between closely related rickettsiae may control rickettsial establishment in arthropods (dual infections in arthropod vectors are rare and have not yet been observed in individual fleas) (*34*). Fleas, however, feed intermittently on different hosts and thus may acquire multiple bacterial strains and pass these to their progeny transovarially (*2*). Our finding of *R. felis* with either *B. clarridgeiae* or *B. quintana* in fleas suggests that dual infections may occur in humans infected by flea feces. We did not, however, find fleas infected with several species of *Bartonella* although cats may be bacteremic with two different species. We believe that the techniques we used should have been sensitive enough to detect such coinfections, and that competition between two *Bartonella* species may result in one species being eliminated and the flea becoming infected with the other *Bartonella* species. Further studies are indicated to confirm this possibility.

Finally, we report, for the first time, the presence of *W. pipientis* in fleas, which we found while seeking *Ehrlichia* using *Anaplasmataceae-*specific primers. This bacteria is known to be an endosymbiont of several arthropods, primarily insects, and it has a role in parthenogenesis (*35*). Any effects that it might have on fleas, however, are unknown.

In summary, our study has provided evidence that cat fleas are commonly infected by *R. felis* and *Bartonella* species in France. Further, we have shown for the first time that *B. quintana* may infect fleas. The description of *B. quintana*-related diseases in patients with histories of contact with fleas indicates the possibility that fleas may be vectors of the organism. Finally, we reported for the first time the presence of *B. koehlerae* outside the United States.
